# Vasovagal Syncope and Pulseless Electrical Activity Cardiac Arrest in Patients With Immunoglobulin Light Chain Cardiac Amyloidosis: A Case Series

**DOI:** 10.7759/cureus.34107

**Published:** 2023-01-23

**Authors:** Brett W Sperry, Ahmed A Harhash, Furha Cossor, Shahzad Raza

**Affiliations:** 1 Department of Cardiology, Saint Luke’s Mid America Heart Institute, Kansas City, USA; 2 Department of Cardiology, University of Vermont, Burlington, USA; 3 Department of Hematology, Saint Luke’s Hospital, Kansas City, USA; 4 Department of Hematology and Medical Oncology, Cleveland Clinic, Cleveland, USA

**Keywords:** amyloidosis, heart failure, cardiomyopathy, multiple myeloma, immunoglobulin light chain

## Abstract

Immunoglobulin light chain (AL) amyloidosis may lead to amyloid fibril deposition into peripheral and autonomic nerves, resulting in resting and orthostatic hypotension. While most patients die from progressive heart failure, the most commonly proposed cardiac rhythm associated with sudden death is pulseless electrical activity (PEA). Herein, we describe four patients with severe AL cardiac amyloidosis who had witnessed cardiac arrest with pulseless electrical activity as a result of vasovagal syncope. Healthcare providers should be aware of severe autonomic dysfunction in cardiac amyloidosis and the potential for an abnormal vasovagal response leading to syncope or death.

## Introduction

Immunoglobulin light chain (AL) amyloidosis is a condition characterized by clonal plasma cells that overproduce the light chain portion of immunoglobulins, which misfold, aggregate, and deposit in various tissues leading to their dysfunction [1]. While the heart and kidneys are the most common organs involved, deposition into peripheral and autonomic nerves can be particularly problematic in some patients and lead to resting and orthostatic hypotension [2]. While most patients die from progressive heart failure, the most commonly proposed cardiac rhythm associated with sudden death is pulseless electrical activity (PEA) [2]. PEA occurs when there is electromechanical dissociation, that is, when cardiac electrical activation occurs without subsequent mechanical contraction. PEA typically occurs as a consequence of another metabolic or mechanical phenomenon, and it is not clear why patients with cardiac amyloidosis frequently die from PEA. Herein, we describe a series of four patients between June 2016 and April 2021 at our institution who exhibited a profound vasovagal response after using the bathroom, leading to PEA cardiac arrest.

## Case presentation

**Figure 1:**
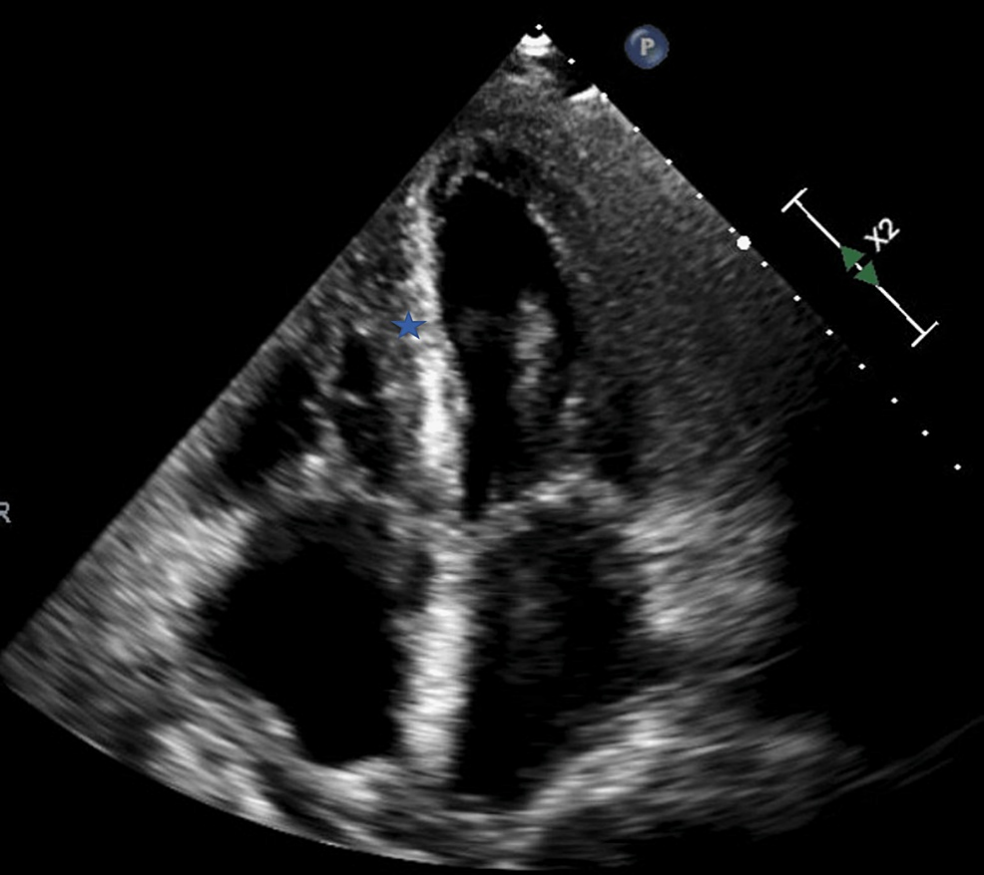
Echocardiogram Apical four-chamber view showing increased left ventricular septal wall thickness (blue star) and bi-atrial enlargement. The ejection fraction here was 27%, and there was severe diastolic dysfunction

Patient 1 was a 49-year-old female with systemic lambda AL amyloidosis with cardiac, renal, and nerve involvement. She was treated with bortezomib and dexamethasone, which led to a very good partial response. Unfortunately, she developed recurrence two years later, requiring the reinitiation of cyclophosphamide, bortezomib, and dexamethasone. Over this period of time, her ejection fraction was 20%-30%, and her septal thickness was 1.5 cm (Figure 1). The patient was admitted with a heart failure exacerbation and treated with a furosemide infusion at 10 mg/hour. After using the bathroom, she had syncope, hypotension, and a PEA arrest that led to death. Telemetry showed atrial fibrillation that degenerated into slow atrial fibrillation and ventricular pacing at the device’s lower rate limit at the time of the cardiac arrest. The autopsy showed multisystem amyloidosis without an acute primary cause of this event.

**Figure 2:**
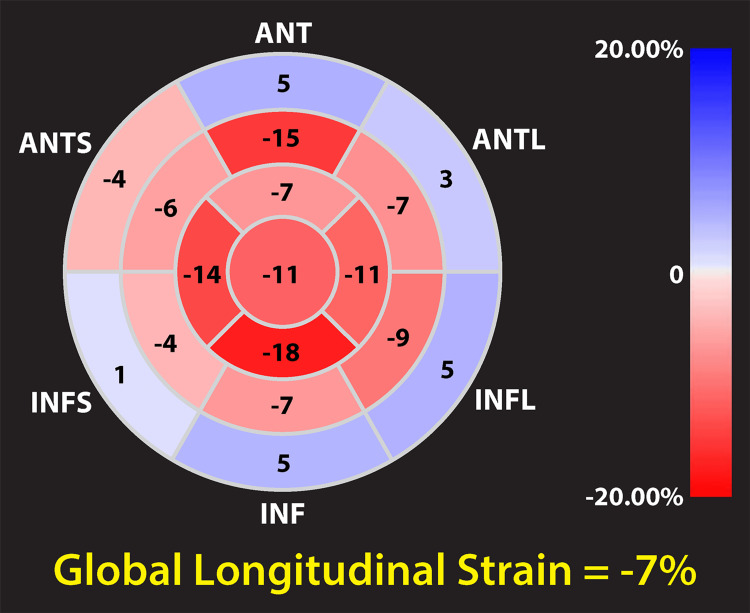
Global longitudinal strain analysis Global longitudinal strain is -7% in this patient, and the polar map shows a characteristic apical sparing pattern consistent with cardiac amyloidosis ANT, anterior; ANTS, anteroseptal; INFS, inferoseptal; INF, inferior; INFL, inferolateral; ANTL, anterolateral

Patient 2 was a 60-year-old male who presented with fluid overload and was found to have newly diagnosed kappa AL amyloidosis with cardiac and renal involvement. Echocardiography showed ejection fraction of 36%, septal thickness of 1.3 cm, and reduced global longitudinal strain with a characteristic apical sparing pattern (Figure 2) [3]. He required inpatient hemodialysis due to anasarca and acute kidney injury. After a 20-day hospitalization, he had syncope and PEA arrest while walking back from the bathroom after an unsuccessful attempt at having a bowel movement and did not survive efforts at resuscitation. Telemetry showed normal sinus rhythm progressing to bradycardia and then asystole.

**Figure 3:**
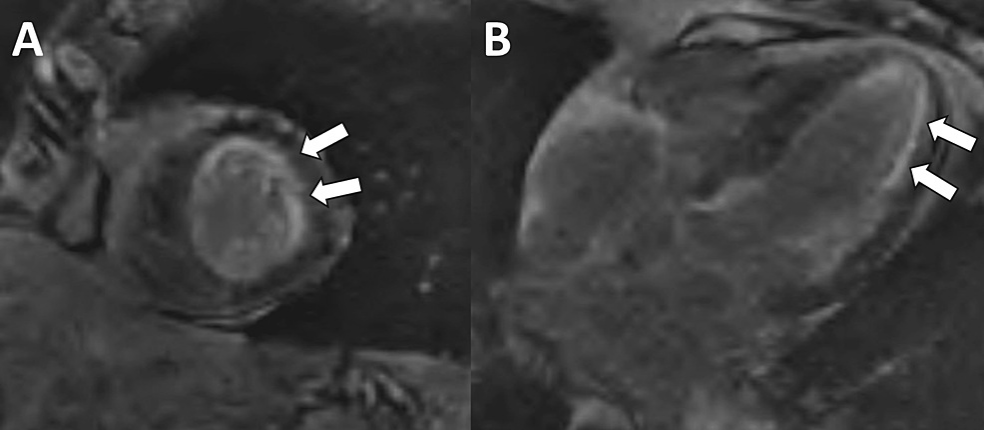
Cardiac magnetic resonance imaging (MRI) A diffuse subendocardial late gadolinium enhancement pattern is noted in the short axis (A) and four-chamber (B) images on this cardiac MRI. The arrows point to the white subendocardial stripe of late gadolinium enhancement in the anterolateral wall

Patient 3 was a 77-year-old female with a history of heart block requiring a pacemaker who presented with newly diagnosed acute heart failure, and lambda AL amyloidosis with cardiac and renal involvement was diagnosed. Echocardiogram showed ejection fraction of 35% and septal thickness of 1.8 cm, and cardiac magnetic resonance imaging (MRI) showed diffuse subendocardial late gadolinium enhancement consistent with amyloidosis (Figure 3). She was initiated on a furosemide infusion without adequate diuresis, and the infusion was increased to 20 mg/hour, and intravenous chlorothiazide was added. Right heart catheterization demonstrated right atrial pressure of 18 mmHg, wedge pressure of 35 mmHg, and cardiac index of 1.98 L/minute/m^2^. The following day, after standing up from having a bowel movement, she syncopized and did not survive efforts at resuscitation. Telemetry initially showed atrial sensing and ventricular pacing, which degenerated into atrial and ventricular pacing at the device’s lower rate limit.

**Figure 4:**
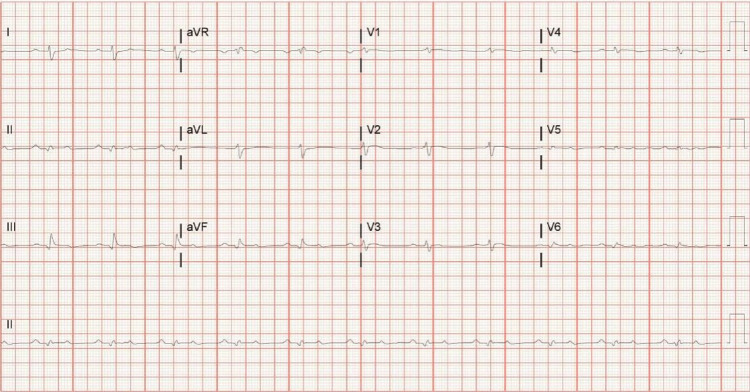
Electrocardiogram A 12-lead electrocardiogram demonstrating normal sinus rhythm with diffuse low voltage, incomplete right bundle block, and a pseudoinfarct pattern in anterior and inferior leads aVR, augmented vector right; aVL, augmented vector left; aVF, augmented vector foot

Patient 4 was a 59-year-old male with newly diagnosed acute heart failure and pleural effusions and was diagnosed with lambda AL cardiac amyloidosis. Electrocardiogram showed sinus rhythm with first-degree atrioventricular (AV) block and diffusely low voltage (Figure 4). Echocardiography showed ejection fraction of 55% with septal thickness of 1.3 cm, and right heart catheterization showed right atrial pressure of 11 mmHg, wedge pressure of 32 mmHg, and cardiac index of 1.99 L/minute/m^2^. He was initiated on a furosemide infusion, which was increased to 20 mg/hour due to inadequate diuresis. He was initiated on bortezomib and dexamethasone while in the hospital. After getting up from having a bowel movement, he developed syncope and PEA arrest. After several rounds of CPR, he had return of spontaneous circulation and survived until hospital discharge 18 days later. He was clinically stable in office follow-up six days after discharge with normal electrolyte levels but had sudden cardiac death at home the following day.

## Discussion

**Figure 5:**
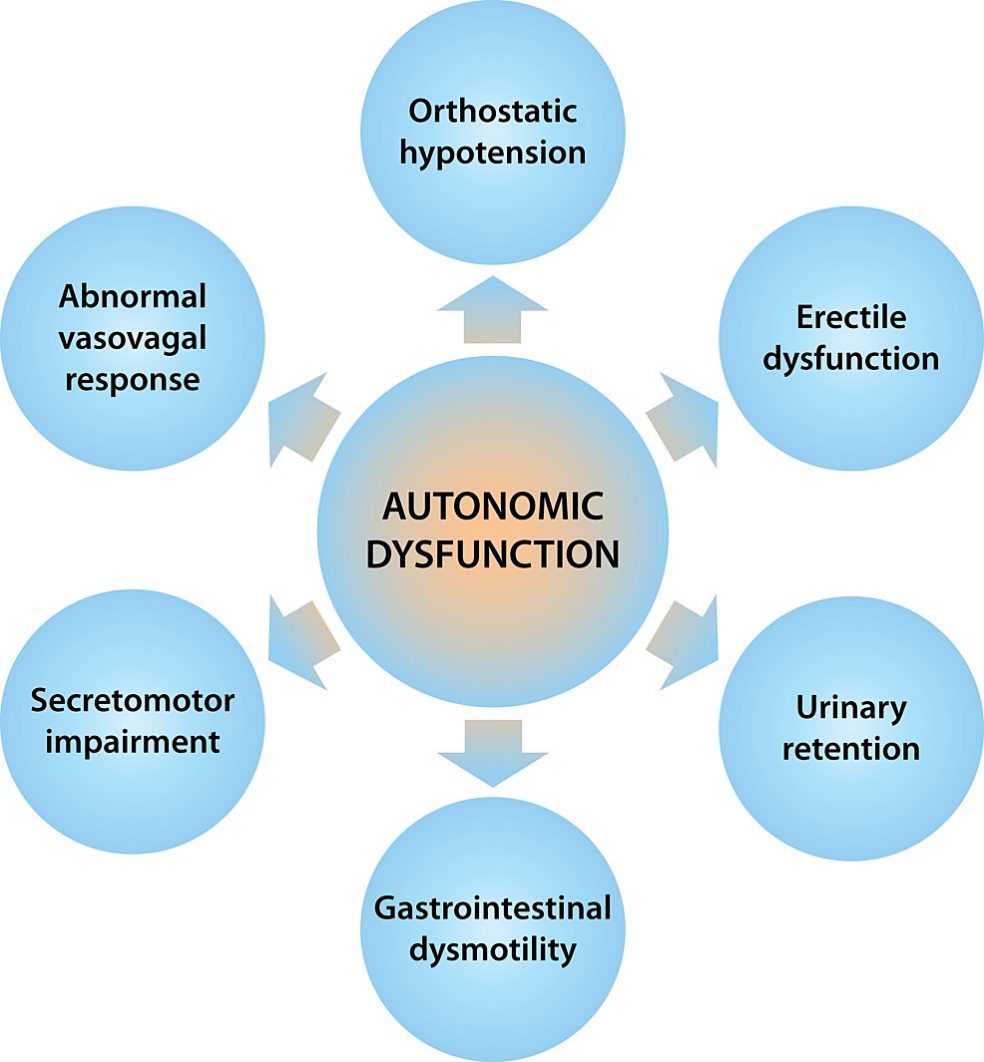
Autonomic dysfunction in amyloidosis The sequelae of autonomic dysfunction seen in patients with AL amyloidosis include orthostatic hypotension, abnormal vasovagal response, secretomotor impairment, gastrointestinal dysmotility, urinary retention, and erectile dysfunction

These cases demonstrate syncope and sudden cardiac death in patients with AL amyloidosis likely related to neurocardiogenic syncope/vasovagal episodes in the setting of severe autonomic dysfunction. AL amyloidosis commonly affects the autonomic nervous system, which may manifest as orthostatic hypotension, gastrointestinal dysmotility, secretomotor impairment, erectile dysfunction, and urinary retention (Figure 5) [4]. The mechanism of syncope in these cases likely relates to cardioinhibitory and vasodepressive responses to the Valsalva maneuver. Of note, all patients were admitted with acute heart failure and were undergoing aggressive diuresis due to anasarca at the time of PEA arrest. Additionally, all patients were receiving high-dose furosemide infusions, which may lead to hypotension and decreased preload through prostaglandin release apart from its diuretic effects [5]. Unfortunately, high-dose intravenous loop diuretics are often needed in these patients with acute heart failure and massive anasarca.

The largest study to describe the mode of death in patients with cardiac amyloidosis included 139 mortalities, 62% of which were cardiovascular. Of these, about two-thirds were from worsening heart failure and 23% from sudden death [6]. A study of 20 consecutive patients implanted loop recorders in those with newly diagnosed severe AL amyloidosis and symptoms of syncope/presyncope; eight patients who died in the hospital had device interrogation that all showed PEA, which was preceded by bradycardia (including complete heart block) a median of one hour before death. The authors proposed that prophylactic pacemakers should be studied in patients with severe AL cardiac amyloidosis. We did not note any prolonged bradyarrhythmias in our cohort, and two patients died of PEA arrest despite having a pacemaker. There is some controversy about whether or not pacemakers are helpful in patients with vasovagal syncope, though randomized controlled trials have shown a reduction in syncopal episodes [7,8].

## Conclusions

In conclusion, we present four patients with severe AL cardiac amyloidosis who had witnessed cardiac arrest with PEA as a result of vasovagal syncope. Healthcare providers should be aware of severe autonomic dysfunction in cardiac amyloidosis and the potential for an abnormal vasovagal response leading to syncope or death. Precipitating factors such as aggressive diuresis and rapid fluid shifts, vasodilator medications, and straining should be avoided.
